# Organic Electronics from Nature: Computational Investigation of the Electronic and Optical Properties of the Isomers of Bixin and Norbixin Present in the Achiote Seeds

**DOI:** 10.3390/molecules27072138

**Published:** 2022-03-25

**Authors:** Igo Tôrres Lima, Josiel da Silva Crispim, Olimpio Pereira de Sá Neto, Rafael Timóteo de Sousa Júnior, Luiz Antônio Ribeiro Júnior, Demétrio Antonio da Silva Filho

**Affiliations:** 1 Coordenação do Bacharelado Interdisciplinar em Ciência e Tecnologia, Campus Dom Delgado, Universidade Federal do Maranhão, São Luís 65080-805, MA, Brazil; igo.torres@ufma.br; 2 Programa de Pós-Graduação em Química, Universidade Estadual do Piauí, Rua João Cabral 2231, Teresina 64002-150, PI, Brazil; josielkimico@gmail.com; 3 Coordenação de Ciência da Computação, Universidade Estadual do Piauí, Parnaiba 65202-220, PI, Brazil; olimpiopereira@phb.uespi.br; 4 Instituto de Física, Universidade Federal do Rio de Janeiro, CP 68.528, Rio de Janeiro 21941-972, RJ, Brazil; 5 Departamento de Engenharia Elétrica, Campus Darcy Ribeiro, Universidade de Brasília, Brasília 70919-970, DF, Brazil; desousa@unb.br; 6 Instituto de Física, Campus Darcy Ribeiro, Universidade de Brasília, Brasília 70919-970, DF, Brazil; ribeirojr@unb.br

**Keywords:** natural dye-sensitized solar cells, organic electronic, achiote seeds

## Abstract

Organic compounds have been employed in developing new green energy solutions with good cost-efficiency compromise, such as photovoltaics. The light-harvesting process in these applications is a crucial feature that still needs improvements. Here, we studied natural dyes to propose an alternative for enhancing the light-harvesting capability of photovoltaics. We performed density functional theory calculations to investigate the electronic and optical properties of the four natural dyes found in achiote seeds (*Bixa orellana* L.). Different DFT functionals, and basis sets, were used to calculate the electronic and optical properties of the bixin, norbixin, and their trans-isomers (molecules present in *Bixa orellana* L.). We observed that the planarity of the molecules and their similar extension for the conjugation pathways provide substantially delocalized wavefunctions of the frontier orbitals and similar values for their energies. Our findings also revealed a strong absorption peak in the blue region and an absorption band over the visible spectrum. These results indicate that *Bixa orellana* L. molecules can be good candidates for improving light-harvesting in photovoltaics.

## 1. Introduction

Alternatives for silicon-based photovoltaics, such as dye-sensitized solar cells (DSSCs) [1,2,3], organic photovoltaics [4,5,6], and perovskite solar cells [7], have not yet reached the desired maturity concerning their physicochemical stability and efficiency. A problem that still limits their efficiency is the low light-harvesting capability (LHC) [8]. Particularly, DSSCs emerged as technically and economically credible alternatives [1,2,3,9,10,11,12,13,14,15]. Their working principle considers increasing the LHC by including a layer of molecular dyes to potentialize the exciton creation. These excitons may be further dissociated into free charge carriers, improving the efficiency of the photovoltaic effect [1,2,3]. Since the advent of DSSCs, the search for new dyes that can enhance their LHC has received much attention [3].

One of the critical materials in DSSC is the sensitizer. Ruthenium-based DSSC complexes show high efficiency and excellent stability, implying potential practical applications [1,16]. However, ruthenium dyes are not suitable for environmentally friendly photovoltaic systems. Ruthenium is expensive and environmentally hazardous, and ruthenium-based compounds are highly toxic and carcinogenic. When these compounds are heated in the presence of air, they form ruthenium tetroxide, which is a highly volatile and toxic compound that damages the eyes and upper respiratory system [17]. Due to this reason, natural dyes are considered in DSSC as substitutes for ruthenium. Natural dyes are easily and safely extracted from plants, not requiring complex synthesis or toxicity tests to be used in DSSCs. Moreover, they have a low cost of synthesis and are environmentally friendly.

So far, natural dyes in DSSCs have shown overall conversion efficiencies below 1%. Several natural dyes such as betalains [18,19], anthocyanins [20,21], and carotenes [22] have been used as sensitizers in DSSCs. Recently, a considerable sensitization activity using the natural dyes extracted from Pastinaca sativa and Beta vulgaris was achieved [22]. It was found that the betaxanthin and betacyanin dyes, each with absorptions at different wavelengths, helped the DSSC to capture photons of two different energies. Short-circuit photocurrent density (JSC) and the open-circuit voltage (VOC) for a DSSC using Pastinaca sativa as a sensitizer are 0.42 V and 7.2 mA/cm${}^{2}$, respectively.

Among the extensive class of natural dyes, achiote (*Bixa orellana* L.) is a plant commonly found in South and Central America. After being crushed, its seeds serve as condiments and food coloring. Moreover, the pigments in these seeds (especially the bixin and norbixin) are used in the textile, cosmetic, and pharmaceutical industries [23,24]. Importantly, these molecules were considered in a DSSC application [25].

**Figure 1:**
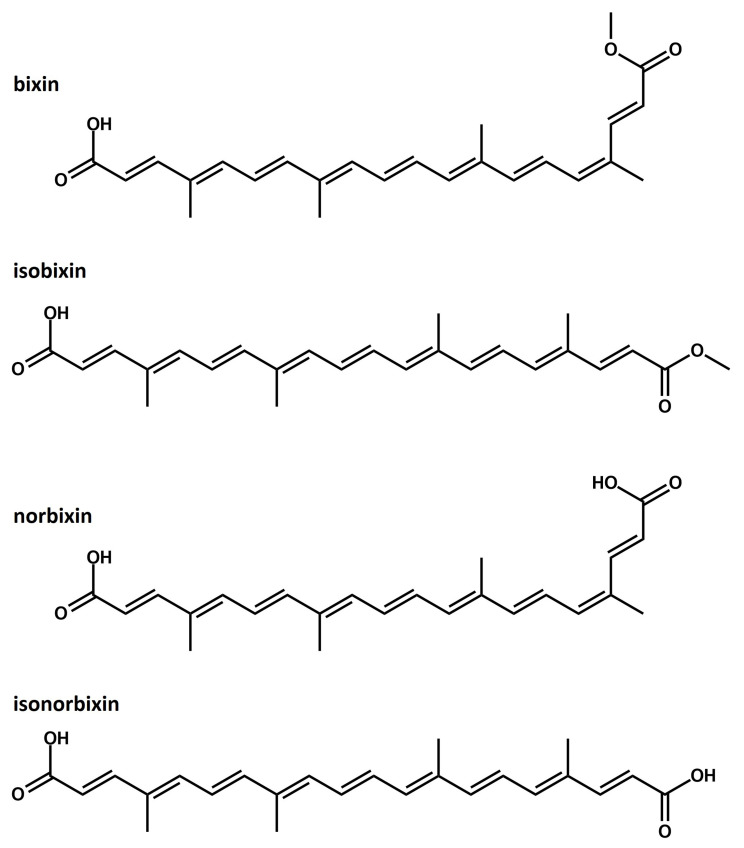
Schematic representation of the lattice structure of bixin, isobixin, norbixin, and isonorbixin.

The bixin molecule (see Figure 1) represents nearly 80% of the pigments in the seed, with the molecular formula $C_{25}H_{30}O_{4}$. Bixin has a carboxylic acid functional group at one end and an ester group at the other. These groups are separated by nine conjugated double bonds, serving as excellent receptors of free radicals and substituting methyl [26]. Commonly, bixin has geometric isomerism *Z* in the sixteenth carbon and isomerism *E* in the rest of the chain [27]. The isomeric structure in which all the carbons of the chain are in isomerism *E*, named trans-bixin or isobixin (see Figure 1), may be formed with the pigment extraction process [27].

Norbixin, in turn, has the molecular formula $C_{24}H_{28}O_{4}$. It has a similar structure to bixin but with a carboxylic acid group in each extremity. This configuration is responsible for its anionic property and hydrosoluble character [28]. Similar to bixin, norbixin also presents isomerism *Z* in its conjugated chain. Submitting the norbixin to controlled heating is a way of producing the trans-norbixin or isonorbixin (see Figure 1). Although these pigments have been used in developing DSSCs [25], some of their optoelectronic and structural properties remain under-investigated.

Efficiency in photovoltaics is strictly related to the exciton dissociation mechanism, and a crucial aspect in the operation of these devices is the distinct nature of the optically excited states [29,30]. In inorganic photovoltaic devices, light absorption directly yields free charge carriers. Conversely, this process in organic-based materials leads to the formation of delocalized electron and hole states, which are coupled due to the strong electron-lattice interactions forming an exciton. The exciton binding energy in organic materials is about 500 meV, ten times higher concerning inorganic materials [29]. Although bixin and norbixin pigments were used to fabricate a DSSC, we address their nonlinear optical and excited-state properties (crucial aspects in the photovoltaic operation), which remain un-discussed. Moreover, we point to the best computational approach to estimate these properties with values comparable to the experimental data. Herein, we analyze through DFT and time-dependent DFT (TDDFT) calculations of the structural and optoelectronic properties of four molecules present in the achiote (*Bixa orellana* L.): bixin, isobixin, norbixin, and isonorbixin, for possible applications of these natural dyes in photovoltaics. The computational protocol employed here considers different DFT functionals and basis sets used to estimate crucial optical parameters of these dyes, such as the vertical transition energies, wavelengths, oscillator strengths, and transition dipole moments. The bond-length alternation (BLA), frontier molecular orbitals, nonlinear optical properties, and absorption spectra were also obtained. The BLA provides information on conjugated oligomers once the extension of the conjugation pathway in their backbone is an important parameter associated with the mobility of the charge carriers.

## 2. Computational Details

DFT is a widely used tool for electronic structure calculations as it provides reliable information without a high computational cost. In the literature, there are several forms of development for the exchange and correlation functional, which seeks a real potential value of the interaction between the electrons of a system, for example, the generalized gradient approximation (GGA), a very popular approximation which, depending on the electron density and its gradient, describes the exchange and correlation energies [31]. The introduction of a term of a second-order derivative of density and/or kinetic energy density as additional degrees of freedom gives rise to another approximation, the meta-GGA [32]. Furthermore, there are also hybrid functionals, a combination of formulations of the Hartree-Fock exchange functional with exchange approximations and correlations used in DFT, such as GGA and meta-GGA. This form of blending improves the performance of energies in some situations and the different forms of this approach are related to the parameter used in the formulations. The Lee–Yang–Parr three-parameter Becke exchange and correlation functional (B3LYP) is a GGA hybrid, composed of the Hartree–Fock exchange functional (HF) and the GGA exchange and correlation approximation [33]. In addition, B3LYP has a version that includes a long-range fix, which addresses the Coulomb attenuating (CAM) method. This functional is known as CAM-B3LYP [34]. Another highlighted functional is Minnesota 2006 (M06), consisting of a meta-GGA hybrid type exchange and correlation functional. It is reasonable in evaluations of proton affinity in conjugated polyene chain and a good description of the π−π stacking interaction [35].

To obtain the optimized molecular geometries, we employed DFT calculations considering three different functionals, i.e., B3LYP, M06, and CAMB3LYP, with the 6-31+G(d,p) basis set [33,34,35,36]. We also performed the geometry optimization of the molecules presented in Figure 1 using these functionals and the 6-31G and 6-31G(d,p) basis sets. All the calculations considered molecules in the gas phase. The polarized continuum model (PCM) was used to include molecules in solution with chloroform.

Low-lying singlet excited states were evaluated at the optimized geometries using time-dependent density functional theory (TDDFT) [37]. The optical absorption profiles were simulated through convolution of the vertical transition energies with the Gaussian functions by a full width at half maximum (FWHM) equal to 0.37 eV (3000 cm${}^{-1}$). We adopted FWHM = 0.37 eV for all peaks since it is the standard value used in the literature, presenting a good track record [38,39]. All calculations were performed using the Gaussian 09 (Revision D.01) suite [40].

## 3. Results

### 3.1. Structural and Electronic Properties

**Figure 2:**
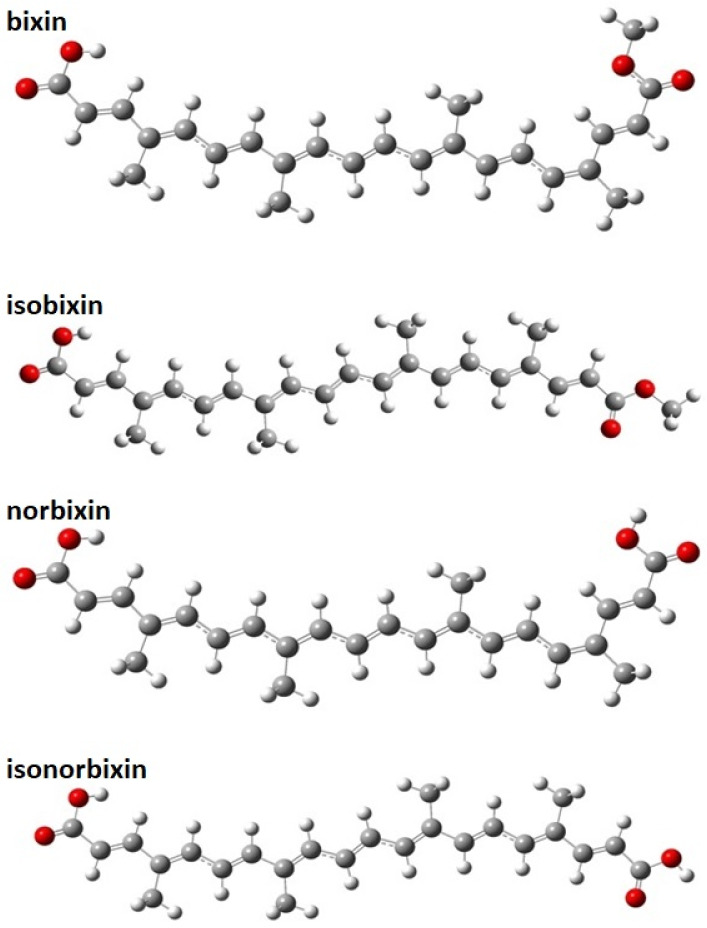
Optimized geometries of the bixin, isobixin, norbixin, and isonorbixin in chloroform solution. These geometries were obtained within the framework of the CAM-B3LYP/6-31+G(d,p) level of theory.

We begin our discussion by presenting the geometric properties of the molecular dyes studied here. Figure 2 illustrates their optimized structures. As a general trend, we observed that the molecules, in both chloroform solution and gas-phase cases, present nearly planar lattice configurations with small torsion angles (about 1–2 degrees) in the edges. Such a signature for the lattice arrangement allows for the wavefunction delocalization on the π-conjugated backbone. All the molecules showed similar extensions of conjugation, i.e., nine carbon double bonds C=C on the π-conjugated backbone. According to the earlier studies [41,42], these findings indicate that the electronic and optical properties of the dyes in Figure 1 tend to present similar behavior.

**Figure 3:**
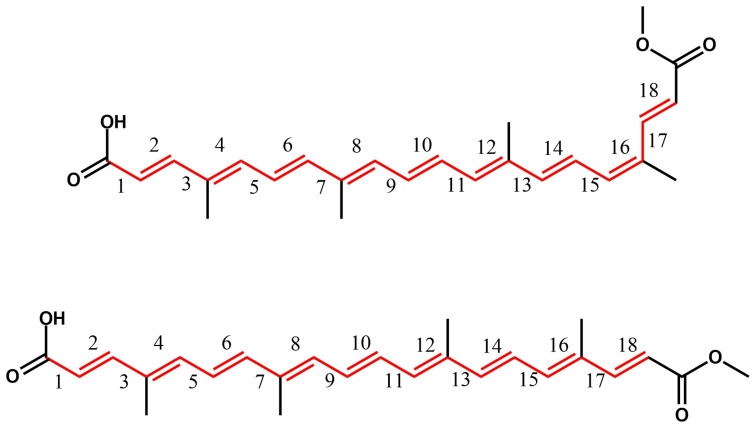
Examined single and double bonds to determine the BLA value.

As mentioned above, BLA is a crucial geometric parameter related to the electronic energy gap [43,44]. BLA is defined as $(R_{\mathrm{single}}-R_{\mathrm{double}})/N$, where $R_{\mathrm{single}}$, $R_{\mathrm{double}}$, and N denote single bond length, double bonds length, and the number of the single-double bond pairs in a π-delocalized system, respectively [44]. Here, we used the BLA values to realize possible changes in the bond length configuration of the dyes. In this way, Figure 3 shows the examined bonds, and Table 1 and Table A1, Table A2, Table A3, Table A4, Table A5 and Table A6 (Appendix A) show bond lengths and BLA values of the π-conjugated backbone for the dyes in gas phase and chloroform solution. For each DFT functional, we observed that both bond lengths and BLA values are similar among the dyes, and the solvent effect can be observed on the reduction of the BLA values when contrasted with the gas phase molecules. CAM-B3LYP provides higher single-bond lengths and lower double-bond lengths concerning the results obtained by employing B3LYP and M06. Consequently, results from CAM-B3LYP present higher BLA values. The total BLA values increased, in sequence, from B3LYP, M06, to CAM-B3LYP, indicating that the higher Hartree–Fock (HF) contribution on the DFT functional leads to higher BLA values. This behavior also impacted the HOMO-LUMO energy gap, as shown later.

**Figure 4:**
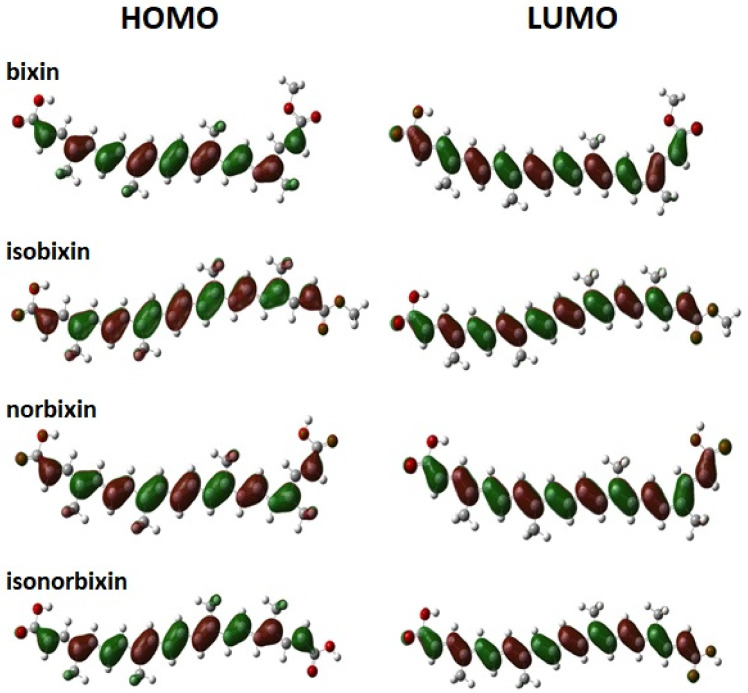
Schematic representation of the frontier molecular orbitals HOMO (left) and LUMO (right) of the molecular dyes in chloroform solution. These results were obtained by employing the CAM-B3LYP/6-31+G(d,p) level of theory.

**Figure A1:**
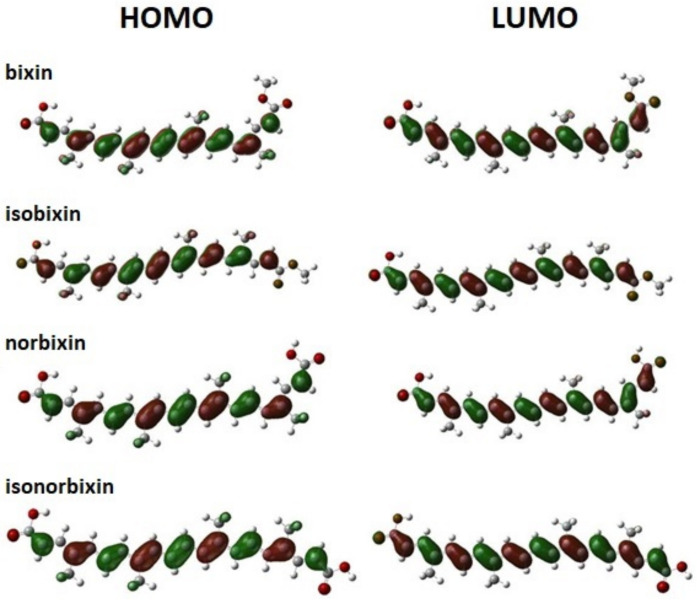
Schematic representation of the frontier molecular orbitals HOMO (left) and LUMO (right) of the molecular dyes in chloroform solution. These results were obtained by employing the B3LYP/6-31+G(d,p) level of theory.

**Figure A2:**
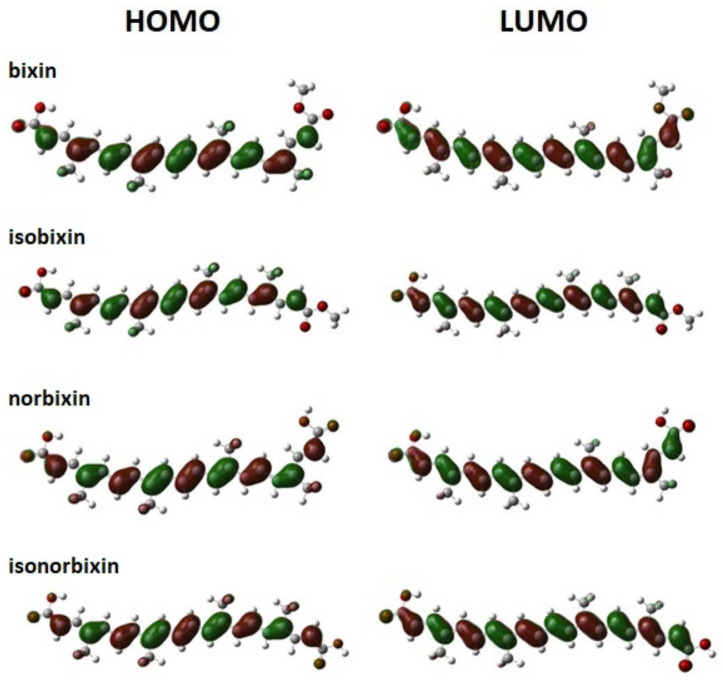
Schematic representation of the frontier molecular orbitals HOMO (left) and LUMO (right) of the molecular dyes in chloroform solution. These results were obtained by employing the M06/6-31+G(d,p) level of theory.

**Figure A3:**
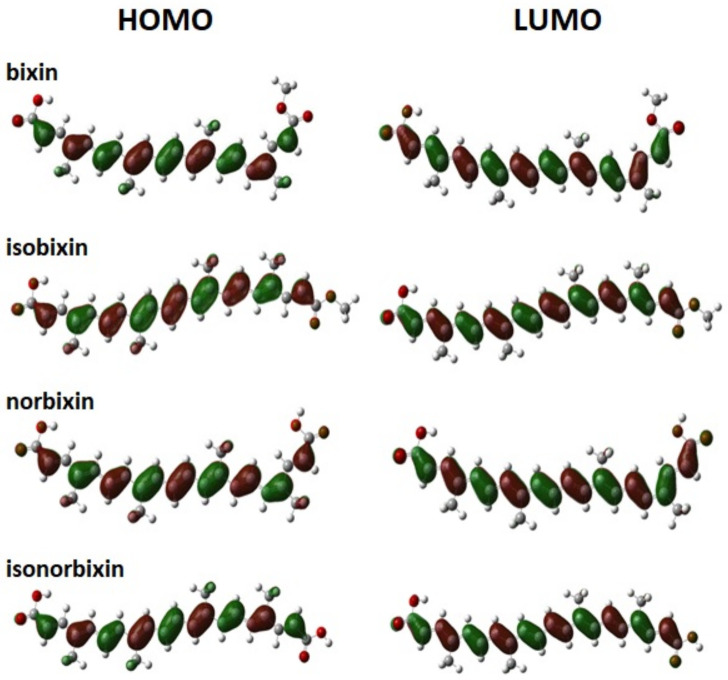
Schematic representation of the frontier molecular orbitals HOMO (left) and LUMO (right) of the molecular dyes in gas phase. These results were obtained by employing the CAM-B3LYP/6-31+G(d,p) level of theory.

Figure 4 and Figure A1, Figure A2 and Figure A3 (see Appendix A) illustrate the HOMO and LUMO wavefunctions of the dyes in the chloroform solution and gas-phase cases. One can note that the frontier molecular orbitals widely delocalized on the π-conjugated backbone. Moreover, no impediment to electronic mobility along the π-conjugated chain was realized. This feature aggregates a metallic character to the polyenic systems since π-electrons of the conjugated chains are not part of a particular bond between atoms, which allows the charge to move along the chain freely [45].

According to Koopman’s theorem, the HOMO energy is the first approximation to the potential of molecular ionization [46]. By analogy, the LUMO energy is an approximation for the electron affinity. In this context, Table 2 and Table A7, Table A8, Table A9, Table A10, Table A11 and Table A12 (see Appendix A) show the energies of the frontier molecular orbitals (MOs) and HOMO-LUMO gap energies of the dyes in the gas phase and chloroform solution. One can note a slight variation of the frontier MOs energies and gap energy for the same functional. We observed differences in the gap energy values for each DFT functional, which increased from B3LYP, M06, to CAM-B3LYP in sequence. These differences are related to the HF contribution since high HF contributions to the DFT functionals induce higher gap energy values. We also note an interplay between BLA and the electronic gap, where an increase in the BLA values leads to an increase in gap energy values. In general, for all DFT functionals and basis sets used, the cis conformation presented gap energy values higher than the trans conformation, i.e., the trans conformation is energetically more stable than its cis analog.

### 3.2. Nonlinear Optical Properties

The nonlinear optical (NLO) response in conjugated organic molecules can be optimized by varying the BLA values as proposed by Marder et al. [47]. It is essential to choose an appropriate basis set for the accurate description of NLO properties [48,49,50]. In this context, we investigate the electric properties of the isomers both in the gas-phase and chloroform solution to see how these properties were impacted both by the BLA behavior and by the choice of basis set. The analyzed quantities were the normal experimentally measured values, i.e., the dipole moment magnitude $\mu =\sqrt{\mu_{x}^{2}+\mu_{y}^{2}+\mu_{z}^{2}}$, the average linear polarizability $\alpha =\frac{\alpha_{xx}+\alpha_{yy}+\alpha_{zz}}{3}$, and the vector component of the first hyperpolarizability $\beta_{vec}=\sum_{i}\frac{\beta_{i}\mu_{i}}{\mu }$; [i=x,y,z], where $\beta_{i}=\sum_{k}\beta ikk$; [k=x,y,z].

Table 3 and Table A13, Table A14, Table A15 and Table A16 (see Appendix A) show the absolute values of obtained electric quantities for different DFT functionals and basis sets. Here, we observed that the values of the μ, α, and $\beta_{vec}$ obtained with the CAM-B3LYP functional are smaller than B3LYP and M06. The exception occurred in isonorbixin with B3LYP that presented smaller values of μ and $\beta_{vec}$ compared to CAM-B3LYP and M06. As CAM-BLYP provided the highest values of BLA (see Table 1), the findings indicate that higher values of BLA had lower values of μ, α, and $\beta_{vec}$. This relationship is in agreement with the work of Labidi et al. for transhexatriene [51]. In addition, the isobixin presented the highest values of μ and α, and bixin presented the highest values of $\beta_{vec}$.

Furthermore, the 6-31+G(d,p) basis set provided the highest values of α followed in descending order by 6-31G and 6-31G(d,p) basis sets, so the inclusion of the diffuse function on the basis set induced increasing α values. On the other hand, this behavior was not observed in μ and $\beta_{vec}$. The highest values of μ and $\beta_{vec}$ of isomers in the gas-phase were provided by the 6-31G basis set. However, there were some cases where the 6-31+G(d,p) basis set provided the highest values of μ and $\beta_{vec}$ of isomers in chloroform solution. Furthermore, the findings of μ and $\beta_{vec}$ with the 6-31G(d,p) basis set were the lowest compared to the 6-31G and 6-31+G(d,p) basis set. We concluded the analysis of the NLO properties of isomers by observing that the solvent effect caused an increase of μ, α, and $\beta_{vec}$.

### 3.3. Excited States Properties

**Figure 5:**
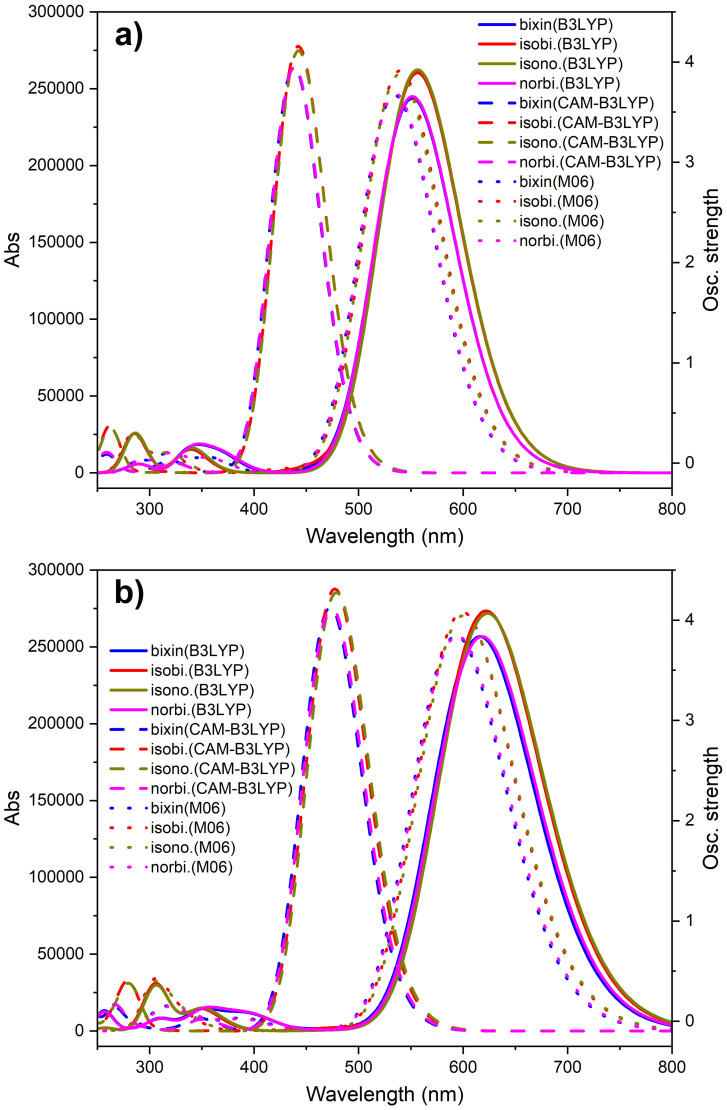
Absorption spectra of the molecular dyes in (**a**) gas phase and (**b**) chloroform solution. These results were obtained by employing the 6-31+G(d,p) basis set.

**Figure A4:**
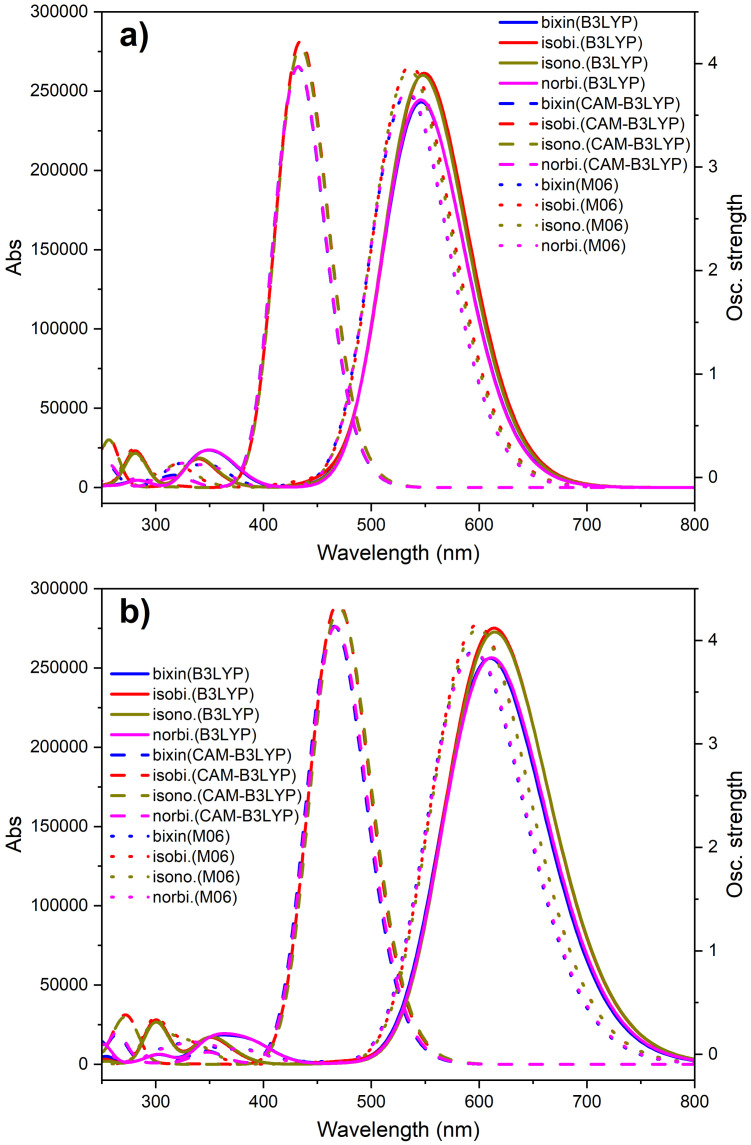
Absorption spectra of molecular dyes in (**a**) gas phase and (**b**) chloroform solution. These results were obtained by employing the 6-31G basis set.

**Figure A5:**
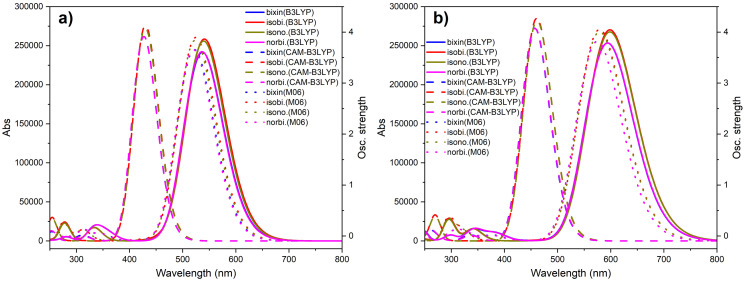
Absorption spectra of molecular dyes in (**a**) gas phase and (**b**) chloroform solution. These results were obtained by employing the 6-31G(d,p) basis set.

We now turn to the description of the low-lying excited states and optical properties of the molecular dyes. Here, we used Gaussian convolution of the wavelength to obtain the absorption spectra of these molecules and then compare the theoretical results of the UV-Vis absorption peak position with the experimental values. Figure 5, Figure A4 and Figure A5 (see Appendix A) show the absorption spectra of the molecules obtained by the Gaussian convolution of the vertical transitions with the FWHM = 0.37 eV (3000 cm${}^{-1}$). One can see that the solvent induces a slight shift in the absorption spectra to higher wavelengths considering the absorption bands. For each case, i.e., gas phase or chloroform solution with the same DFT functional and basis set, all molecules presented maximum absorption peaks in the same region, which can be associated with the same extension of backbone conjugation and close gap energies.

Calculations with different DFT functionals revealed a shift in the absorption band positions to higher wavelengths from CAM-B3LYP, M06, to B3LYP, in sequence. We associate the increase of the gap energy with the decrease of the wavelength (analogously, an increase in the vertical energy). The relationship between the BLA values and the position of the absorption bands can be understood as follows: an increase in the average BLA value implies a decrease in the overlap between atomic orbitals, consequently leading the absorption bands to shift to higher energies (lower wavelengths).

Finally, Table 4, Table A17 and Table A18 (see Appendix A) show the values of the vertical transition energy ($E_{01}$), the wavelength of the maximum absorption peak ($\lambda_{01}$), oscillator strength (*f*), and transition dipole moment ($\mu_{01}$). In all cases, we observed that the transition dipole moment was mainly on the conjugated backbone, i.e., along the x-direction, and isobixin and isonorbixin presented higher values about $\mu_{01}$. The experimental data showed that the maximum peaks of bixin, isobixin, norbixin, and isonorbixin were in the blue region, i.e., 470, 476, 468, and 475 nm, respectively [52]. From these data, we conclude that the results obtained with the CAM-B3LYP functional and 6-31+G(d,p) basis set presented a better description of the optical properties. We can attribute this result to the higher HF contribution on the CAM-B3LYP functional compared to the others, combined with the inclusion of the diffuse and polarization functions on the basis set that is generally more appropriate to describe polyene systems.

## 4. Conclusions

In summary, we employed DFT and TD-DFT calculations to study the geometrical and optoelectronic properties of bixin and norbixin isomers. These molecules are present in the achiote seeds, a plant found in tropical America. Since they present a clear UV-Vis absorption spectrum, they can be good candidates for developing novel DSSCs. The DFT and TD-DFT calculations were conducted within the framework of three different functionals (B3LYP, CAM-B3LYP, and M06) and basis sets (6-31+G(d,p), 6-31G(d,p), and 6-31G).

As a general trend, we observed that these molecules in chloroform solution and gas-phase presented almost planar lattice configurations with small torsion angles in the edges. Such a lattice arrangement allows wavefunction delocalization on the π-conjugated backbone. Moreover, their similar extension in the conjugation pathway leads to close values for their MO energies. The HOMO-LUMO gap energy values increased from the B3LYP, M06, to CAM-B3LYP levels of theory, in the sequence as a response for increasing HF contribution to the DFT functional.

In the optical properties study, we observed that the increase of the HF contribution is reflected in the shift of the bands to lower wavelengths (or higher energies). The absorption bands of the molecules in the chloroform solution were slightly shifted to a higher wavelength concerning the gas phase. We also obtained the vertical transition energies, wavelengths, oscillator strengths, and transition dipole moments. Here, we observed that the transition dipole moments for all the molecular dyes were aligned with the molecular axis, and the comparison with the experimental data showed that the CAM-B3LYP functional, with the 6-31+G(d,p) basis set, provided a better description of the optical properties.

It is important to stress that bixin represents the main carotenoid found in the achiote seeds. In addition, the absorption peak position was comparable for both Z-isomer (bixin) and E-isomer (isobixin), which indicates that light capture and exciton formation tend to be similar in the two isomers.

This work provided a benchmark on the computational methodologies for the electronic and optical characterization of natural dyes, which is absent in the literature. Although bixin and norbixin do not perform better than other molecules recently reported in the literature for some photovoltaic applications, they are still worth investigating. These molecules are abundant in Tropical America (composed only of developing countries) and have easy extraction. These crucial features can aggregate in the final product a good cost-benefit relationship, which is attractive when it comes to manufacturing of organic-based optoelectronic devices and their possible commercialization in developing countries.

## Figures and Tables

**Table 1:** Total BLA values (Å) in the gas phase (ϵ = 1.00) and chloroform (ϵ = 4.71) solution, which were determined by employing the 6-31+G(d,p) basis set.

Functional	Bixin	Isobixin	Norbixin	Isonorbixin
		gas		
B3LYP	0.077	0.076	0.076	0.074
CAM-B3LYP	0.101	0.100	0.100	0.100
M06	0.080	0.079	0.079	0.079
		chloroform		
B3LYP	0.073	0.072	0.073	0.072
CAM-B3LYP	0.098	0.097	0.098	0.097
M06	0.077	0.076	0.076	0.076

**Table 2:** Frontier HOMO/LUMO MOs and gap energy values for the dyes in chloroform solution. These results were obtained by employing the 6-31+G(d,p) basis set.

Functional	HOMO (eV)	LUMO (eV)	Gap (eV)
bixin			
B3LYP	−5.221	−3.073	2.148
CAM-B3LYP	−6.360	−1.853	4.507
M06	−5.428	−2.889	2.538
isobixin			
B3LYP	−5.220	−3.099	2.121
CAM-B3LYP	−6.353	−1.879	4.474
M06	−5.426	−2.918	2.508
norbixin			
B3LYP	−5.244	−3.104	2.141
CAM-B3LYP	−6.379	−1.879	4.499
M06	−5.447	−2.915	2.532
isonorbixin			
B3LYP	−5.249	−3.134	2.115
CAM-B3LYP	−6.376	1.911	4.465
M06	−5.450	−2.948	2.503

**Table 3:** Dipole moment (μ), average linear polarizability (α), and vector component of the first ($\beta_{vec}$) hyperpolarizability of the dyes in gas phase and chloroform solution. These results were obtained by employing the 6-31+G(d,p) basis set.

Functional	μ (Debye)	α ($10^{-24}$ esu)	β ($10^{-30}$ esu)
bixin in gas phase			
B3LYP	5.81	116.14	122.65
CAM-B3LYP	5.10	89.31	67.83
M06	5.73	110.94	104.58
isobixin in gas phase			
B3LYP	6.94	121.86	104.85
CAM-B3LYP	6.42	92.34	47.23
M06	6.75	116.07	86.1
norbixin in gas phase			
B3LYP	5.91	114.35	87.68
CAM-B3LYP	5.36	87.66	51.87
M06	5.77	109.37	78.67
isonorbixin in gas phase			
B3LYP	2.81	119.66	4.56
CAM-B3LYP	5.25	90.01	24.85
M06	5.48	113.08	51.21
bixin in chloroform solution			
B3LYP	6.91	160.85	371.31
CAM-B3LYP	5.73	112.98	144.21
M06	6.72	151.08	284.73
isobixin in chloroform solution			
B3LYP	8.29	168.52	262.47
CAM-B3LYP	7.52	116.22	76.87
M06	7.92	158.06	195.13
norbixin in chloroform solution			
B3LYP	7.06	159.6	276.48
CAM-B3LYP	6.13	111.52	117.24
M06	6.94	149.76	218.19
isonorbixin in chloroform solution			
B3LYP	6.66	166.67	141.87
CAM-B3LYP	6.28	114.40	37.08
M06	6.53	155.75	111.75

**Table 4:** $S_{0}\to S_{1}$ vertical transition energies ($E_{01}$), wavelength ($\lambda_{01}$), oscillator strength (*f*), and transition dipole moments ($\mu_{01}$). These results were obtained by employing the 6-31+G(d,p) basis set.

Molecule	$E_{01}$ (eV)	$\lambda_{01}$ (nm)	*f*	$\mu_{01}$ (Debye)			
				x	y	z	tot
gas/B3LYP							
bixin	2.249	551.32	3.363	−19.844	0.109	−0.003	19.845
isobixin	2.229	556.16	3.591	−20.536	1.552	0.017	20.594
norbixin	2.249	551.35	3.379	19.883	−0.605	0.004	19.892
isonorbixin	2.229	556.23	3.620	−20.635	1.344	0	20.679
chloroform/B3LYP							
bixin	2.013	616.07	3.542	−21.528	0.061	−0.013	21.528
isobixin	1.994	621.91	3.773	−22.258	−1.707	0	22.323
norbixin	2.006	618	3.542	21.553	−0.652	0.001	21.563
isonorbixin	1.989	623.28	3.75	22.232	−1.477	0	22.281
gas/CAM-B3LYP							
bixin	2.831	437.99	3.642	−18.393	0.708	0.003	18.407
isobixin	2.805	441.98	3.83	−18.837	−2.170	0.028	18.961
norbixin	2.828	438.39	3.643	18.380	−1.165	0.012	18.417
isonorbixin	2.804	442.23	3.791	18.767	−1.972	0.026	18.870
chloroform/CAM-B3LYP							
bixin	2.624	472.53	3.802	−19.520	−0.762	0	19.535
isobixin	2.599	477.03	3.970	19.914	−2.393	0.025	20.057
norbixin	2.616	473.92	3.800	19.515	−1.299	0.006	19.558
isonorbixin	2.591	478.54	3.939	19.890	2.166	0.018	20.008
gas/M06							
bixin	2.320	534.5	3.391	−19.617	0.313	−0.009	19.619
isobixin	2.298	539.43	3.612	20.260	−1.819	0.027	20.342
norbixin	2.317	535.1	3.403	19.650	−0.774	0.006	19.665
isonorbixin	2.301	538.76	3.578	20.168	−1.626	0.025	20.234
chloroform/M06							
bixin	2.090	593.12	3.547	−21.135	0.316	−0.032	21.137
isobixin	2.069	599.32	3.762	21.789	−2.031	0.026	21.884
norbixin	2.086	594.51	3.547	21.144	0.881	−0.017	21.163
isonorbixin	2.065	600.4	3.732	21.741	1.800	0.016	21.816

## Data Availability

The data presented in this study are available in the main article and Appendix A.

## Appendix A

**Table A1:** Single-double bonds and local BLA values (Å) of the molecular dyes in gas phase. These results were obtained by employing the B3LYP/6-31+G(d,p) level of theory.

	bixin			isobixin		
bonds	single	double	local BLA	single	double	local BLA
$b_{1}-b_{2}$	1.479	1.356	0.123	1.479	1.356	0.123
$b_{3}-b_{4}$	1.451	1.371	0.079	1.451	1.371	0.080
$b_{5}-b_{6}$	1.434	1.367	0.067	1.434	1.367	0.067
$b_{7}-b_{8}$	1.443	1.376	0.067	1.443	1.376	0.067
$b_{9}-b_{10}$	1.429	1.370	0.059	1.429	1.371	0.059
$b_{11}-b_{12}$	1.430	1.375	0.054	1.429	1.376	0.053
$b_{13}-b_{14}$	1.445	1.366	0.079	1.444	1.367	0.077
$b_{15}-b_{16}$	1.438	1.372	0.065	1.435	1.371	0.064
$b_{17}-b_{18}$	1.451	1.355	0.097	1.450	1.354	0.095
	**norbixin**			**isonorbixin**		
bonds	single	double	local BLA	single	double	local BLA
$b_{1}-b_{2}$	1.479	1.356	0.123	1.465	1.356	0.109
$b_{3}-b_{4}$	1.451	1.371	0.080	1.450	1.372	0.078
$b_{5}-b_{6}$	1.434	1.367	0.067	1.434	1.367	0.067
$b_{7}-b_{8}$	1.443	1.376	0.067	1.443	1.376	0.067
$b_{9}-b_{10}$	1.429	1.370	0.059	1.429	1.371	0.059
$b_{11}-b_{12}$	1.430	1.375	0.054	1.429	1.376	0.053
$b_{13}-b_{14}$	1.445	1.366	0.079	1.443	1.367	0.076
$b_{15}-b_{16}$	1.437	1.373	0.064	1.434	1.372	0.062
$b_{17}-b_{18}$	1.450	1.356	0.094	1.448	1.356	0.092

**Table A2:** Single-double bonds and local BLA values (Å) of the molecular dyes in gas phase. These results were obtained by employing the CAM-B3LYP/6-31+G(d,p) level of theory.

	bixin			isobixin		
bonds	single	double	local BLA	single	double	local BLA
$b_{1}-b_{2}$	1.480	1.344	0.137	1.480	1.344	0.137
$b_{3}-b_{4}$	1.457	1.356	0.101	1.457	1.356	0.101
$b_{5}-b_{6}$	1.444	1.351	0.092	1.444	1.351	0.092
$b_{7}-b_{8}$	1.453	1.358	0.095	1.453	1.358	0.095
$b_{9}-b_{10}$	1.441	1.353	0.088	1.441	1.353	0.088
$b_{11}-b_{12}$	1.441	1.358	0.083	1.441	1.358	0.083
$b_{13}-b_{14}$	1.454	1.350	0.104	1.454	1.351	0.103
$b_{15}-b_{16}$	1.446	1.356	0.090	1.444	1.356	0.088
$b_{17}-b_{18}$	1.457	1.343	0.114	1.455	1.343	0.112
	**norbixin**			**isonorbixin**		
bonds	single	double	local BLA	single	double	local BLA
$b_{1}-b_{2}$	1.481	1.344	0.137	1.481	1.344	0.137
$b_{3}-b_{4}$	1.457	1.356	0.101	1.457	1.356	0.101
$b_{5}-b_{6}$	1.444	1.351	0.093	1.444	1.351	0.093
$b_{7}-b_{8}$	1.453	1.358	0.095	1.453	1.358	0.095
$b_{9}-b_{10}$	1.441	1.353	0.088	1.441	1.353	0.088
$b_{11}-b_{12}$	1.441	1.358	0.083	1.441	1.358	0.083
$b_{13}-b_{14}$	1.454	1.350	0.104	1.453	1.351	0.102
$b_{15}-b_{16}$	1.446	1.357	0.089	1.444	1.356	0.087
$b_{17}-b_{18}$	1.456	1.344	0.112	1.454	1.344	0.110

**Table A3:** Single-double bonds and local BLA values (Å) of the molecular dyes in gas phase. These results were obtained by employing the M06/6-31+G(d,p) level of theory.

	bixin			isobixin		
bonds	single	double	local BLA	single	double	local BLA
$b_{1}-b_{2}$	1.474	1.349	0.124	1.474	1.349	0.124
$b_{3}-b_{4}$	1.446	1.364	0.082	1.446	1.364	0.082
$b_{5}-b_{6}$	1.431	1.360	0.071	1.431	1.360	0.071
$b_{7}-b_{8}$	1.439	1.368	0.071	1.439	1.368	0.071
$b_{9}-b_{10}$	1.426	1.362	0.064	1.426	1.362	0.063
$b_{11}-b_{12}$	1.427	1.367	0.059	1.426	1.368	0.058
$b_{13}-b_{14}$	1.441	1.358	0.082	1.439	1.359	0.080
$b_{15}-b_{16}$	1.434	1.365	0.069	1.431	1.364	0.068
$b_{17}-b_{18}$	1.446	1.349	0.098	1.444	1.348	0.096
	**norbixin**			**isonorbixin**		
bonds	single	double	local BLA	single	double	local BLA
$b_{1}-b_{2}$	1.474	1.349	0.125	1.474	1.349	0.124
$b_{3}-b_{4}$	1.446	1.364	0.082	1.446	1.364	0.082
$b_{5}-b_{6}$	1.430	1.360	0.071	1.431	1.360	0.071
$b_{7}-b_{8}$	1.439	1.368	0.071	1.439	1.368	0.071
$b_{9}-b_{10}$	1.426	1.363	0.063	1.426	1.362	0.064
$b_{11}-b_{12}$	1.426	1.368	0.059	1.426	1.368	0.058
$b_{13}-b_{14}$	1.440	1.359	0.081	1.439	1.359	0.080
$b_{15}-b_{16}$	1.433	1.365	0.068	1.431	1.364	0.067
$b_{17}-b_{18}$	1.445	1.350	0.095	1.443	1.349	0.094

**Table A4:** Single-double bonds and local BLA values (Å) of the molecular dyes in chloroform solution. These results were obtained by employing the B3LYP/6-31+G(d,p) level of theory.

	bixin			isobixin		
bonds	single	double	local BLA	single	double	local BLA
$b_{1}-b_{2}$	1.469	1.359	0.110	1.469	1.359	0.111
$b_{3}-b_{4}$	1.447	1.374	0.073	1.447	1.374	0.073
$b_{5}-b_{6}$	1.433	1.369	0.064	1.433	1.369	0.064
$b_{7}-b_{8}$	1.443	1.377	0.065	1.442	1.377	0.065
$b_{9}-b_{10}$	1.430	1.371	0.058	1.429	1.371	0.058
$b_{11}-b_{12}$	1.430	1.376	0.054	1.429	1.377	0.052
$b_{13}-b_{14}$	1.445	1.367	0.078	1.444	1.368	0.076
$b_{15}-b_{16}$	1.437	1.374	0.063	1.434	1.373	0.061
$b_{17}-b_{18}$	1.449	1.357	0.093	1.448	1.357	0.091
	**norbixin**			**isonorbixin**		
bonds	single	double	local BLA	single	double	local BLA
$b_{1}-b_{2}$	1.469	1.359	0.110	1.470	1.359	0.111
$b_{3}-b_{4}$	1.448	1.374	0.074	1.448	1.374	0.074
$b_{5}-b_{6}$	1.433	1.369	0.064	1.433	1.369	0.065
$b_{7}-b_{8}$	1.443	1.377	0.066	1.443	1.377	0.065
$b_{9}-b_{10}$	1.430	1.371	0.058	1.429	1.371	0.058
$b_{11}-b_{12}$	1.430	1.377	0.053	1.429	1.377	0.052
$b_{13}-b_{14}$	1.445	1.367	0.078	1.443	1.368	0.075
$b_{15}-b_{16}$	1.436	1.375	0.062	1.434	1.374	0.060
$b_{17}-b_{18}$	1.448	1.358	0.090	1.446	1.358	0.088

**Table A5:** Single-double bonds and local BLA values (Å) of the molecular dyes in chloroform solution. These results were obtained by employing the CAM-B3LYP/6-31+G(d,p) level of theory.

	bixin			isobixin		
bonds	single	double	local BLA	single	double	local BLA
$b_{1}-b_{2}$	1.472	1.346	0.126	1.473	1.346	0.127
$b_{3}-b_{4}$	1.455	1.358	0.097	1.455	1.358	0.097
$b_{5}-b_{6}$	1.443	1.352	0.091	1.443	1.352	0.091
$b_{7}-b_{8}$	1.453	1.359	0.094	1.453	1.359	0.094
$b_{9}-b_{10}$	1.441	1.354	0.087	1.441	1.354	0.087
$b_{11}-b_{12}$	1.442	1.359	0.083	1.441	1.359	0.082
$b_{13}-b_{14}$	1.455	1.351	0.104	1.454	1.352	0.102
$b_{15}-b_{16}$	1.447	1.358	0.089	1.444	1.357	0.087
$b_{17}-b_{18}$	1.456	1.345	0.111	1.454	1.345	0.109
	**norbixin**			**isonorbixin**		
bonds	single	double	local BLA	single	double	local BLA
$b_{1}-b_{2}$	1.472	1.346	0.126	1.473	1.346	0.127
$b_{3}-b_{4}$	1.455	1.358	0.097	1.455	1.357	0.097
$b_{5}-b_{6}$	1.443	1.352	0.091	1.443	1.352	0.091
$b_{7}-b_{8}$	1.453	1.359	0.094	1.453	1.359	0.094
$b_{9}-b_{10}$	1.441	1.354	0.087	1.441	1.354	0.087
$b_{11}-b_{12}$	1.441	1.359	0.083	1.441	1.359	0.082
$b_{13}-b_{14}$	1.455	1.351	0.103	1.453	1.352	0.102
$b_{15}-b_{16}$	1.446	1.358	0.088	1.443	1.358	0.085
$b_{17}-b_{18}$	1.454	1.346	0.108	1.452	1.346	0.106

**Table A6:** Single-double bonds and local BLA values (Å) of the molecular dyes in chloroform solution. These results were obtained by employing the M06/6-31+G(d,p) level of theory.

	bixin			isobixin		
bonds	single	double	local BLA	single	double	local BLA
$b_{1}-b_{2}$	1.465	1.352	0.113	1.465	1.352	0.113
$b_{3}-b_{4}$	1.443	1.366	0.077	1.443	1.366	0.077
$b_{5}-b_{6}$	1.430	1.361	0.068	1.430	1.361	0.069
$b_{7}-b_{8}$	1.439	1.369	0.070	1.439	1.369	0.070
$b_{9}-b_{10}$	1.427	1.363	0.063	1.426	1.363	0.063
$b_{11}-b_{12}$	1.427	1.368	0.059	1.426	1.369	0.058
$b_{13}-b_{14}$	1.441	1.359	0.081	1.439	1.360	0.079
$b_{15}-b_{16}$	1.433	1.366	0.067	1.431	1.365	0.065
$b_{17}-b_{18}$	1.445	1.351	0.094	1.443	1.351	0.092
	**norbixin**			**isonorbixin**		
bonds	single	double	local BLA	single	double	local BLA
$b_{1}-b_{2}$	1.465	1.352	0.113	1.465	1.352	0.113
$b_{3}-b_{4}$	1.443	1.366	0.077	1.443	1.366	0.077
$b_{5}-b_{6}$	1.430	1.361	0.069	1.430	1.361	0.069
$b_{7}-b_{8}$	1.439	1.369	0.070	1.439	1.369	0.070
$b_{9}-b_{10}$	1.427	1.363	0.063	1.426	1.363	0.063
$b_{11}-b_{12}$	1.427	1.368	0.058	1.426	1.369	0.057
$b_{13}-b_{14}$	1.440	1.360	0.081	1.439	1.361	0.078
$b_{15}-b_{16}$	1.433	1.367	0.066	1.430	1.366	0.064
$b_{17}-b_{18}$	1.443	1.352	0.091	1.441	1.352	0.089

**Table A7:** Frontier MOs and gap energy values for the dyes in gas phase. These results were obtained by employing the 6-31G basis set. All values are in electron-Volt (eV).

Molecule	HOMO-2	HOMO-1	HOMO	LUMO	LUMO+1	LUMO+2	Gap
**B3LYP**							
bixin	−6.884	−6.068	−5.116	−2.939	−2.122	−1.333	2.177
isobixin	−6.857	−6.041	−5.116	−2.966	−2.095	−1.252	2.150
norbixin	−6.912	−6.123	−5.170	−2.966	−2.177	−1.388	2.204
isonorbixin	−6.912	−6.095	−5.170	−2.993	−2.177	−1.306	2.177
**CAM-B3LYP**							
bixin	−8.245	−7.320	−6.259	−1.714	−0.898	−0.027	4.544
isobixin	−8.245	−7.293	−6.259	−1.742	−0.898	0.054	4.517
norbixin	−8.299	−7.347	−6.286	−1.742	−0.952	−0.082	4.544
isonorbixin	−8.272	−7.347	−6.286	−1.769	−0.952	0.000	4.517
**M06**							
bixin	−7.184	−6.340	−5.361	−2.830	−2.014	−1.170	2.531
isobixin	−7.184	−6.340	−5.361	−2.857	−1.986	−1.088	2.503
norbixin	−7.211	−6.395	−5.415	−2.857	−2.068	−1.225	2.558
isonorbixin	−7.211	−6.395	−5.415	−2.884	−2.041	−1.143	2.531

**Table A8:** Frontier MOs and gap energy values for the dyes in gas phase. These results were obtained by employing the 6-31G(d,p) basis set. All values are in electron-Volt (eV).

Molecule	HOMO-2	HOMO-1	HOMO	LUMO	LUMO+1	LUMO+2	Gap
**B3LYP**							
bixin	−6.803	−5.987	−5.089	−2.830	−1.959	−1.197	2.259
isobixin	−6.776	−5.987	−5.061	−2.857	−1.959	−1.088	2.204
norbixin	−6.830	−6.041	−5.116	−2.857	−2.014	−1.225	2.259
isonorbixin	−6.830	−6.014	−5.089	−2.884	−1.986	−1.143	2.204
**CAM-B3LYP**							
bixin	−8.191	−7.265	−6.231	−1.578	−0.735	0.136	4.653
isobixin	−8.163	−7.238	−6.231	−1.605	−0.707	0.245	4.626
norbixin	−8.218	−7.293	−6.259	−1.605	−0.762	0.109	4.653
isonorbixin	−8.191	−7.265	−6.259	−1.633	−0.762	0.218	4.626
**M06**							
bixin	−7.102	−6.286	−5.333	−2.694	−1.823	−1.007	2.640
isobixin	−7.075	−6.259	−5.306	−2.694	−1.823	−0.925	2.612
norbixin	−7.129	−6.313	−5.361	−2.721	−1.878	−1.061	2.640
isonorbixin	−7.129	−6.286	−5.361	−2.721	−1.850	−0.952	2.640

**Table A9:** Frontier MOs and gap energy values for the dyes in gas phase. These results were obtained by employing the 6-31+G(d,p) basis set. All values are in electron-Volt (eV).

Molecule	HOMO-2	HOMO-1	HOMO	LUMO	LUMO+1	LUMO+2	Gap
**B3LYP**							
bixin	−7.102	−6.313	−5.388	−3.184	−2.340	−1.578	2.204
isobixin	−7.075	−6.286	−5.361	−3.184	−2.313	−1.469	2.177
norbixin	−7.157	−6.340	−5.415	−3.211	−2.395	−1.660	2.204
isonorbixin	−7.048	−6.231	−5.333	−3.157	−2.286	−1.442	2.177
**CAM-B3LYP**							
bixin	−8.463	−7.538	−6.531	−1.959	−1.088	−0.299	4.572
isobixin	−8.436	−7.510	−6.504	−1.986	−1.088	−0.163	4.517
norbixin	−8.517	−7.592	−6.558	−1.986	−1.170	−0.354	4.572
isonorbixin	−8.490	−7.565	−6.558	−2.014	−1.170	−0.218	4.544
**M06**							
bixin	−7.347	−6.531	−5.578	−2.993	−2.150	−1.388	2.585
isobixin	−7.320	−6.504	−5.551	−3.020	−2.122	−1.279	2.531
norbixin	−7.402	−6.558	−5.606	−3.020	−2.204	−1.442	2.585
isonorbixin	−7.374	−6.558	−5.606	−3.048	−2.177	−1.333	2.558

**Table A10:** Frontier MOs and gap energy values for the dyes in chloroform solution. These results were obtained by employing the 6-31G basis set. All values are in electron-Volt (eV).

Molecule	HOMO-2	HOMO-1	HOMO	LUMO	LUMO+1	LUMO+2	Gap
**B3LYP**							
bixin	−6.776	−5.959	−4.980	−2.857	−2.095	−1.279	2.122
isobixin	−6.776	−5.932	−4.980	−2.884	−2.095	−1.225	2.095
norbixin	−6.803	−5.987	−5.007	−2.884	−2.150	−1.333	2.122
isonorbixin	−6.776	−5.959	−5.007	−2.912	−2.122	−1.252	2.095
**CAM-B3LYP**							
bixin	−8.136	−7.184	−6.123	−1.633	−0.871	0.000	4.490
isobixin	−8.136	−7.184	−6.123	−1.660	−0.898	0.082	4.463
norbixin	−8.163	−7.211	−6.123	−1.660	−0.925	−0.027	4.463
isonorbixin	−8.163	−7.211	−6.123	−1.687	−0.925	0.054	4.435
**M06**							
bixin	−7.075	−6.231	−5.225	−2.748	−1.986	−1.143	2.476
isobixin	−7.075	−6.231	−5.252	−2.776	−1.959	−1.061	2.476
norbixin	−7.102	−6.259	−5.252	−2.776	−2.014	−1.170	2.476
isonorbixin	−7.102	−6.259	−5.252	−2.803	−2.014	−1.088	2.449

**Table A11:** Frontier MOs and gap energy values for the dyes in chloroform solution. These results were obtained by employing the 6-31G(d,p) basis set. All values are in electron-Volt (eV).

Molecule	HOMO-2	HOMO-1	HOMO	LUMO	LUMO+1	LUMO+2	Gap
**B3LYP**							
bixin	−6.694	−5.905	−4.952	−2.748	−1.932	−1.143	2.204
isobixin	−6.694	−5.878	−4.952	−2.776	−1.932	−1.061	2.177
norbixin	−6.721	−5.905	−4.980	−2.776	−1.959	−1.170	2.204
isonorbixin	−6.721	−5.905	−4.980	−2.803	−1.959	−1.088	2.177
**CAM-B3LYP**							
bixin	−8.082	−7.157	−6.123	−1.497	−0.707	0.190	4.626
isobixin	−8.082	−7.129	−6.123	−1.524	−0.707	0.272	4.599
norbixin	−8.109	−7.184	−6.123	−1.524	−0.735	0.163	4.599
isonorbixin	−8.109	−7.157	−6.123	−1.551	−0.735	0.245	4.572
**M06**							
bixin	−6.993	−6.177	−5.197	−2.612	−1.796	−0.980	2.585
isobixin	−6.993	−6.150	−5.197	−2.640	−1.796	−0.898	2.558
norbixin	−7.021	−6.204	−5.225	−2.612	−1.823	−1.007	2.612
isonorbixin	−7.021	−6.177	−5.225	−2.640	−1.823	−0.898	2.585

**Table A12:** Frontier MOs and gap energy values for the dyes in chloroform solution. These results were obtained by employing the 6-31+G(d,p) basis set. All values are in electron-Volt (eV).

Molecule	HOMO-2	HOMO-1	HOMO	LUMO	LUMO+1	LUMO+2	Gap
**B3LYP**							
bixin	−6.973	−6.165	−5.221	−3.073	−2.287	−1.530	2.148
isobixin	−6.965	−6.153	−5.220	−3.099	−2.278	−1.424	2.121
norbixin	−7.003	−6.194	−5.244	−3.104	−2.341	−1.572	2.141
isonorbixin	−6.996	−6.185	−5.249	−3.134	−2.324	−1.466	2.115
**CAM-B3LYP**							
bixin	−8.338	−7.408	−6.360	−1.853	−1.073	−0.251	4.507
isobixin	−8.326	−7.390	−6.353	−1.879	−1.076	−0.126	4.474
norbixin	−8.365	−7.433	−6.379	−1.879	−1.130	−0.294	4.499
isonorbixin	−8.354	−7.419	−6.376	−1.911	−1.123	−0.167	4.465
**M06**							
bixin	−7.223	−6.400	−5.428	−2.889	−2.102	−1.339	2.538
isobixin	−7.215	−6.388	−5.426	−2.918	−2.096	−1.221	2.508
norbixin	−7.248	−6.424	−5.447	−2.915	−2.147	−1.374	2.532
isonorbixin	−7.241	−6.416	−5.450	−2.948	−2.136	−1.257	2.503

**Table A13:** Dipole moment (μ), average linear polarizability (α), and vector component of the first ($\beta_{vec}$) hyperpolarizability of the dyes in gas phase. These results were obtained by employing the 6-31G basis set.

DFT-Functional	μ(Debye)	$\alpha \;\left(10^{-24}\;\mathrm{esu}\right)$	$\beta_{\mathrm{vec}}\;\left(10^{-30}\;\mathrm{esu}\right)$
**bixin**			
B3LYP	6.23	108.91	122.63
CAM-B3LYP	5.51	82.24	69.87
M06	6.26	105.7	110.09
**isobixin**			
B3LYP	6.96	114.44	99.48
CAM-B3LYP	6.50	85.14	45.94
M06	7.06	110.67	87.54
**norbixin**			
B3LYP	6.34	107.06	93.68
CAM-B3LYP	5.75	80.6	56.05
M06	6.33	104.08	85.86
**isonorbixin**			
B3LYP	5.67	111.21	62.25
CAM-B3LYP	5.47	82.71	28.56
M06	5.83	107.5	57.52

**Table A14:** Dipole moment (μ), average linear polarizability (α), and vector component of the first ($\beta_{vec}$) hyperpolarizability of the dyes in chloroform solution. These results were obtained by employing the 6-31G basis set.

DFT-Functional	μ(Debye)	$\alpha \;\left(10^{-24}\;\mathrm{esu}\right)$	$\beta_{\mathrm{vec}}\;\left(10^{-30}\;\mathrm{esu}\right)$
**bixin**			
B3LYP	7.16	150.63	332.61
CAM-B3LYP	6.04	103.28	132.25
M06	7.03	145.28	286.75
**isobixin**			
B3LYP	7.89	158.01	219.69
CAM-B3LYP	7.34	106.54	66.06
M06	7.99	151.95	184.02
**norbixin**			
B3LYP	7.32	149.53	266.03
CAM-B3LYP	6.39	101.78	110.87
M06	7.26	143.84	229.14
**isonorbixin**			
B3LYP	6.52	155.39	133.3
CAM-B3LYP	6.30	104.4	38.54
M06	6.70	149.01	116.7

**Table A15:** Dipole moment (μ), average linear polarizability (α), and vector component of the first ($\beta_{vec}$) hyperpolarizability of the dyes in gas phase. These results were obtained by employing the 6-31G(d,p) basis set.

DFT-Functional	μ(Debye)	$\alpha \;\left(10^{-24}\;\mathrm{esu}\right)$	$\beta_{\mathrm{vec}}\;\left(10^{-30}\;\mathrm{esu}\right)$
**bixin**			
B3LYP	5.17	106.29	85.36
CAM-B3LYP	4.64	81.33	47.88
M06	5.1	102.34	76.80
**isobixin**			
B3LYP	6.08	111.55	71.24
CAM-B3LYP	5.73	84.13	31.17
M06	6.05	106.72	60.45
**norbixin**			
B3LYP	5.29	104.30	65.79
CAM-B3LYP	4.85	79.57	38.94
M06	5.28	100.11	61.7
**isonorbixin**			
B3LYP	5.10	108.27	44.22
CAM-B3LYP	4.93	81.63	19.26
M06	5.19	103.52	41.55

**Table A16:** Dipole moment (μ), average linear polarizability (α), and vector component of the first ($\beta_{vec}$) hyperpolarizability of the dyes in chloroform solution. These results were obtained by employing the 6-31G(d,p) basis set.

DFT-Functional	μ(Debye)	$\alpha \;\left(10^{-24}\;\mathrm{esu}\right)$	$\beta_{\mathrm{vec}}\;\left(10^{-30}\;\mathrm{esu}\right)$
**bixin**			
B3LYP	5.88	143.81	225.45
CAM-B3LYP	5.06	100.93	87.75
M06	5.74	136.84	189.44
**isobixin**			
B3LYP	6.90	150.53	142.5
CAM-B3LYP	6.52	103.95	39.71
M06	6.87	142.58	111.24
**norbixin**			
B3LYP	6.10	142.13	179.24
CAM-B3LYP	5.39	99.19	75.02
M06	5.99	135.01	156.21
**isonorbixin**			
B3LYP	5.88	147.65	85.7
CAM-B3LYP	5.73	101.63	22.20
M06	5.98	139.67	71.32

**Table A17:** $S_{0}\to S_{1}$ vertical transition energies ($E_{01}$), wavelength ($\lambda_{01}$), oscillator strength (*f*), and transition dipole moments ($\mu_{01}$). These results were obtained by employing the 6-31G basis set.

Molecule	$E_{01}$ (eV)	$\lambda_{01}$ (nm)	*f*	$\mu_{01}$ (Debye)			
				x	y	z	tot
**B3LYP**							
gas							
bixin	2.270	546.16	3.353	19.722	−0.151	0.012	19.722
isobixin	2.258	549.01	3.601	20.441	−1.426	−0.006	20.490
norbixin	2.272	545.74	3.373	19.763	−0.613	−0.004	19.772
isonorbixin	2.264	547.67	3.585	20.383	−1.244	0.007	20.421
chloroform							
bixin	2.032	610.11	3.533	21.397	−0.130	0.037	21.397
isobixin	2.020	613.73	3.796	22.190	−1.575	−0.020	22.246
norbixin	2.028	611.47	3.539	21.427	0.669	−0.011	21.438
isonorbixin	2.018	614.26	3.763	22.115	−1.361	0.006	22.157
**CAM-B3LYP**							
gas							
bixin	2.872	431.72	3.663	−18.310	0.769	−0.010	18.326
isobixin	2.856	434.19	3.883	18.809	2.067	−0.019	18.922
norbixin	2.870	431.95	3.664	−18.295	1.200	0.001	18.334
isonorbixin	2.855	434.22	3.832	−18.704	1.882	−0.018	18.799
chloroform							
bixin	2.663	465.52	3.813	19.397	−0.852	0.022	19.416
isobixin	2.644	468.89	4.017	19.871	2.272	−0.016	20.000
norbixin	2.657	466.56	3.810	−19.383	1.347	0.014	19.430
isonorbixin	2.639	469.9	3.973	−19.805	2.072	−0.013	19.913
**M06**							
gas							
bixin	2.325	533.2	3.426	−19.695	0.351	−0.016	19.699
isobixin	2.313	536.1	3.669	−20.368	1.710	−0.017	20.440
norbixin	2.325	533.37	3.440	19.725	−0.796	−0.004	19.741
isonorbixin	2.317	535.06	3.631	−20.256	1.526	−0.015	20.313
chloroform							
bixin	2.086	594.31	3.592	21.290	−0.361	0.033	21.293
isobixin	2.073	598.23	3.826	21.968	−1.888	0.013	22.049
norbixin	2.083	595.17	3.589	21.282	−0.890	−0.023	21.301
isonorbixin	2.071	598.7	3.787	21.879	−1.678	0.007	21.943

**Table A18:** $S_{0}\to S_{1}$ vertical transition energies ($E_{01}$), wavelength ($\lambda_{01}$), oscillator strength (*f*), and transition dipole moments ($\mu_{01}$). These results were obtained by employing the 6-31G(d,p) basis set.

Molecule	$E_{01}$ (eV)	$\lambda_{01}$ (nm)	*f*	$\mu_{01}$ (Debye)			
				x	y	z	tot
**B3LYP**							
gas							
bixin	2.309	536.94	3.334	19.500	−0.174	0.004	19.500
isobixin	2.293	540.64	3.565	20.170	−1.574	−0.015	20.232
norbixin	2.312	536.23	3.344	19.505	−0.601	0.003	19.514
isonorbixin	2.299	539.26	3.531	−20.061	−1.392	−0.017	20.109
chloroform							
bixin	2.088	593.88	3.497	21.002	−0.174	0.007	21.003
isobixin	2.072	598.31	3.731	21.700	−1.775	−0.013	21.773
norbixin	2.087	594.22	3.497	20.998	−0.680	−0.003	21.009
isonorbixin	2.072	598.29	3.693	−21.606	−1.569	−0.014	21.663
**CAM-B3LYP**							
gas							
bixin	2.905	426.73	3.612	−18.078	0.748	−0.001	18.093
isobixin	2.886	429.67	3.809	−18.517	2.167	0.028	18.643
norbixin	2.906	426.62	3.611	−18.052	1.150	−0.008	18.089
isonorbixin	2.887	429.42	3.757	−18.403	1.989	−0.028	18.510
chloroform							
bixin	2.713	457.01	3.756	−19.074	0.847	0.023	19.093
isobixin	2.692	460.61	3.934	−19.467	2.424	0.020	19.617
norbixin	2.710	457.46	3.752	−19.048	1.309	−0.022	19.093
isonorbixin	2.689	461.06	3.892	−19.396	2.228	−0.027	19.523
**M06**							
gas							
bixin	2.377	521.67	3.380	−19.348	−0.358	−0.001	19.351
isobixin	2.364	524.58	3.595	19.930	−1.831	−0.024	20.014
norbixin	2.382	520.42	3.376	19.301	−0.776	−0.003	19.316
isonorbixin	2.368	523.49	3.547	−19.790	−1.656	−0.026	19.859
chloroform							
bixin	2.158	574.47	3.527	−20.741	−0.399	0.003	20.745
isobixin	2.141	579	3.737	21.336	−2.069	−0.011	21.436
norbixin	2.158	574.61	3.523	20.718	−0.885	0.004	20.737
isonorbixin	2.142	578.88	3.695	−21.231	−1.871	−0.022	21.314
